# Empowering Maneuvers: Boldly Mobilizing Military Medical Research and Training within the Kingdom of Saudi Arabia to Safeguard a Nation and its People.

**DOI:** 10.12688/f1000research.151754.1

**Published:** 2024-06-10

**Authors:** Yasser Mandourah, Richard Mottershead, Nafi Alonaizi, Hasan Alriaini, Essam Burhan, Nabeel Al-Yateem

**Affiliations:** 1 Military Medical Services, Riyadh, Saudi Arabia; 2 Department of Nursing, University of Sharjah, Sharjah, Sharjah, United Arab Emirates; 3 Ministry of Defense, Joint Command Forces, Riyadh, Saudi Arabia

**Keywords:** Medical Research, Health Training, National Strategies, Digital Health, Humanitarian Response

## Abstract

In 2023 Alkhathami and colleagues from the Prince Sultan Military College of Health Sciences highlighted the Kingdom of Saudi Arabia’s transformative upgrades across various sectors, notably including enhancements to the healthcare system, and called for action to extend these upgrades to the military healthcare field. Prompted by this call to action, the leadership of the military healthcare system swiftly commenced initiatives, acting in less than three months from this pivotal appeal. In January 2023 the first author ordered a decree via the General Directorate of Armed Forces Medical Services of Saudi Arabia, in collaboration with US central Command and international partners, to host the 3rd International Conference of Military Medicine. The event graciously welcoming more than 1000 military representatives from 20 participating nations. The Military Medical Conference, fostering a global military community dialogue on the necessity to explore collective capacities to endure and overcome humanitarian challenges, thereby sustaining health, promoting well-being, and nurturing life through strategies that align with the insights of Alkhathami et al. (2023). The response and the need underlined by the original article are discussed by the Major General, staff of the Saudi Military Medical Services and academics from the University of Sharjah.

## Introduction

Alkhathami et al. (2023) underscored the imperative to progress and advance military medical research and training within the Saudi Armed Forces to meet the nation’s research and development needs. Considering the significant financial investments in the Kingdom of Saudi Arabia’s (KSA) Armed Forces ( Trending Economics, 2021), aligning research and training policy and practice is crucial to devising an effective and empowering strategy. This response aims to not only resonate with the original article but also to attest to the evidence of the plan’s progression, supporting and accelerating the military medical services’ contributions towards achieving the Kingdom’s Vision 2030 targets ( Saudi Vision, 2021).

This significant advancement towards these goals showcases the collaboration between Saudi Arabian researchers and international experts, laying a foundation for success through mutual support and comradery—a theme highlighted by the lead author at this year’s military medical conference. However, this response also calls for a continued confidence in leadership and celebrates the knowledge and capabilities of Arab states, drawing upon international partnerships to advance military medical research and training. This ethos underscores the region’s collaborative spirit and dedication to enhancing the medical services’ bespoke needs, ensuring the safety and sovereignty of Saudi Arabia and its neighboring states ( Mottershead & Alonaizi, 2022a). The Ministry of Defense Health Military Medical Services (MODMMS) and the Saudi Ministry of Defense have demonstrated innovation and foresight in enhancing military healthcare services through research and training initiatives, a commitment this response applauds as evidence of the Vision’s blueprint for economic and social success by 2030. This progress is realized within national initiatives to progress the digital health strategy through the progression of digitalization. This method is a comprehensive solution that provides the scalable and interoperable tactical medical architecture required to collect, collate, analyze, and distribute critical medical data across the All-Domain Military Operations. This endeavor spearheaded by the first author and his team is a direct reference to the needs discussed by Alkhathami et al. (2023) in the goal for Saudi Arabia is actively pursue localizing its military industries as part of its Vision 2030 plan, intending to localise 50% of military industries by 2030.

This national project’s digital health strategy will provide documentation through interoperability of medical communications for combat casualty care systems. The authors acknowledge and heed the concerns of Alkhathami et al. (2023), in their belief that traditional clinical medical research, alone is insufficient to meet the specific needs of the military. A comprehensive and solution focused strategy has been mobilized to accelerate the research and training needs of the nation as evidenced with the recent joint Forces conference. This event showcased the digital health strategy and how innovation will now automate and synchronize the collection of medical data to support medical regulating. Digitalization will further integrate and allow bidirectional exchange within existing military satellite networks, inadvertently, enabling the transmission and receiving of medical data via the Ministry of Defense (MoD) communication Network and other secure networks. Crucially important is that it shall provide Situational Awareness (SA) and understanding using a Common Operational Picture (COP) for command systems. The authors highlight how within this research and training initiative, intelligence from the area of operations shall integrate medical devices to provide casualty heat maps, Command and Control and medical evacuation (MERT).

Alkhathami et al. (2023) counsel that there is a lack of comprehensive research and evidence specifically tailored to the military context in Saudi Arabia. The authors advocate forward planning and a collaborative and cohesive response to these opportunities to further advance the achievements of the nation. Mottershead and Alonaizi (2022b) evidence attempts to progress healthcare treatments and therapeutic alliances to empower new health and well-being strategies within the region. The recent conference focusing on a Multinational Medical Response themed on Emergency Disaster Management, Humanitarian Assistance & Relief Operations, Major General Yasser Mandourah, General Director of Armed Forces Medical Services (first author) has sought to combat against a regression in military medicine readiness that occurs between periods of active service. This pattern, referred to as Walkers Dip (2018) results in a diminished ability to respond. MODMMS has therefore shown great insight by creating a strategy to safeguard the lives of young service personnel by maintaining the effectiveness of not only the Saudi but also all participating nations military medical services. This ability resonates with the nation’s executive leadership, acknowledging the valued insight from colleagues such as Alkhathami et al. (2023) and celebrates the nation’s proud ability to listen and respond to the ever-present need for the continuation of knowledge embraced within the solidarity of comradeship.

In conclusion, this article celebrates the progress made in investing in military medical research and training as vital components of the readiness and effectiveness of Saudi military medical services. This agenda promises to enhance care for military personnel, and foster improved training, research, and development. The inherent need to innovate, woven into the fabric of the region’s people, has always necessitated resourcefulness. This empowering capability has been a cornerstone of prosperity and identity for the Kingdom of Saudi Arabia and its people. Finally, the authors advocate for the mobilization towards an agenda that populates research designed to meet the contextualized needs of the military research landscape, creating a rallying call to meet and exceed the ambitions set forth by the Kingdom of Saudi Arabia’s Vision 2030.

## Author contributions

YM and RM conceived the article. YM and RM prepared the first draft of the manuscript. All authors were involved in the revision of the draft manuscript and have agreed the content.
