# Supplementary material for: Empowering Maneuvers: Boldly Mobilizing Military Medical Research and Training within the Kingdom of Saudi Arabia to Safeguard a Nation and its People
Source: F1000Res. 2024 Jun 10;13:613. [Version 1] doi: 10.12688/f1000research.151754.1 (PMC11269971; doi:10.12688/f1000research.151754.1)
Supplement: Peer review history

# Reviewer report: Reviewer response for version 1

**DOI:** 10.5256/f1000research.166427.r291517

**Published:** 2024-07-24

**Authors:** Neil Kitchiner

**Affiliations:** 1 Cardiff University, Cardiff, Wales, UK

Empowering Maneuvers: Boldly Mobilizing Military Medical Research and Training within the Kingdom of Saudi Arabia to Safeguard a Nation and its People.

 The above paper builds on a previous indexing by (Alkhathami et al. 2023) regarding the Kingdom of Saudi Arabia's plans to transform the military healthcare system amongst other sectors. It calls for action to extend these upgrades to the military healthcare field. They hosted an international partners conference who attended the 3rd International Conference of Military Medicine with delegates from over 20 countries attending.

 The indexing highlights Saudi Arabia's national project to enhance it's digital health strategy allowing for medical documentation to flow from the combat arena via combat casualty care systems enhancing the command and control and medical evacuation. The indexing also promotes research designed to meet the above's needs as described in the Kingdom of Saudi Arabia's Vision 2023.

Are arguments sufficiently supported by evidence from the published literature or by new data and results?

Yes

Is the conclusion balanced and justified on the basis of the presented arguments?

Yes

Is the rationale for commenting on the previous publication clearly described?

Yes

Are any opinions stated well-argued, clear and cogent?

Yes

Reviewer Expertise:

Military mental health

I confirm that I have read this submission and believe that I have an appropriate level of expertise to confirm that it is of an acceptable scientific standard.

# Reviewer report: Reviewer response for version 1

**DOI:** 10.5256/f1000research.166427.r291516

**Published:** 2024-07-23

**Authors:** Ali Alhaiti

**Affiliations:** 1 AlMaarefa University, Diriyah, Riyadh, Saudi Arabia

Abstract:

 The authors should not give any citations in the abstract section.

 Are arguments sufficiently supported by evidence from the indexed literature or by new data and results?

 The arguments are partly sufficient because there is a paucity of literature supporting this study. Furthermore, the authors have not stated sufficient data that supports this article. I therefore urge the authors to clearly address this issue to make this article scientifically sound.

 This article is relevant because it addresses the challenges and limitations of the previous article indexed by Alkhathami et al. (2023). Moreover, it led to an international collaboration that laid a foundation via mutual support and camaraderie. The article also yields a positive result by making the Saudi Government invest heavily in military medical research and training. However, the author stated that “it was highlighted how this research and training initiative, intelligence from the area of operations shall integrate medical devices to provide casualty heat maps, Command and Control and medical evacuation”. What did the author highlight?

Are arguments sufficiently supported by evidence from the published literature or by new data and results?

Partly

Is the conclusion balanced and justified on the basis of the presented arguments?

Yes

Is the rationale for commenting on the previous publication clearly described?

Yes

Are any opinions stated well-argued, clear and cogent?

Yes

Reviewer Expertise:

Community Health Nursing

I confirm that I have read this submission and believe that I have an appropriate level of expertise to confirm that it is of an acceptable scientific standard.

## References

[1] : Empowering Maneuvers: Boldly Mobilizing Military Medical Research and Training within the Kingdom of Saudi Arabia to Safeguard a Nation and its People. *F1000Research* .2024;13: 10.12688/f1000research.151754.1 10.12688/f1000research.151754.1 PMC1126997139055882

[2] : Advancing military medical research and training in the Saudi Armed Forces: a call to action. *BMJ Military Health* .2023; 10.1136/military-2023-002528 10.1136/military-2023-002528 PMC1201507637923313
